# Pulmonary Embolism in the Cancer Associated Thrombosis Landscape

**DOI:** 10.3390/jcm11195650

**Published:** 2022-09-25

**Authors:** Géraldine Poenou, Teona Dumitru Dumitru, Ludovic Lafaie, Valentine Mismetti, Elie Ayoub, Cécile Duvillard, Sandrine Accassat, Patrick Mismetti, Marco Heestermans, Laurent Bertoletti

**Affiliations:** 1 Therapeutic and Vascular Medicine Department, University Hospital of Saint Etienne, 42270 Saint Etienne, France; 2 Internal Medicine Department, University Hospital Santa Lucía, 30202 Cartagena, Spain; 3 Health Sciences PhD Program, Universidad Católica de Murcia UCAM, Campus de los Jerónimos nº135, 30107 Guadalupe, Spain; 4 Geriatric Department, University Hospital of Saint Etienne, 42270 Saint Etienne, France; 5 INSERM, U1059, SAINBIOSE, Jean Monnet University, 42023 Saint-Etienne, France; 6 Pneumology Department, University Hospital of Saint Etienne, 42270 Saint Etienne, France; 7 INSERM, CIC-1408, CHU Saint-Etienne, 42055 Saint-Etienne, France; 8 French Blood Establishment Auvergne Rhône Alpes—Scientific Department, 42270 Saint-Etienne, France

**Keywords:** cancer, pulmonary embolism, epidemiology, diagnosis, prognosis, management

## Abstract

In cancer patients, pulmonary embolism (PE) is the second leading cause of death after the cancer itself, most likely because of difficulties in diagnosing the disease due to its nonclassical presentation. The risk of PE recurrence and possibly the case-fatality rate depends on whether the patient presents a symptomatic PE, an unsuspected PE, a subsegmental PE, or a catheter-related PE. Choosing the best therapeutic option is challenging and should consider the risk of both the recurrence of thrombosis and the occurrence of bleeding. The purpose of this review is to provide an overview of the clinical characteristics and the treatment of cancer-associated PE, which could benefit clinicians to better manage the deadliest form of thrombosis associated with cancer. After a brief presentation of the epidemiological data, we will present the current attitude towards the diagnosis and the management of cancer patients with PE. Finally, we will discuss the perspectives of how the medical community can improve the management of this severe medical condition.

## 1. Introduction

Cancer is one of the major acquired risk factors for venous thromboembolism (VTE) with a 4- to 7-fold increased risk to develop the disease [1]. In cancer patients, pulmonary embolism (PE) is the deadliest form of associated thrombosis. In cancer patients, PE represents the second leading cause of death after the cancer itself [2]. Cancer patients are more fragile, and the paucisymptomatic clinical presentation of PE in these individuals makes the diagnosis more difficult. These two conditions favor a prognosis that is known to be inferior to that of noncancer patients, with a non-negligible risk of recurrent PE or of bleeding events. Moreover, the therapeutic arsenal that is used for the treatment or prevention of recurrent PE is either not validated or not without any associated risk in cancer-associated PE. Knowing how to diagnose, treat, and prevent PE is a major concern for the management of cancer patients. Identifying clinical characteristics that separate cancer patients with an increased risk of PE from the low- to medium-risk cancer population is crucial to improve clinical outcomes. Within this review, we aim to (i) outline the epidemiological characteristics of PE in cancer patients, (ii) provide an update of the diagnosis and the prognosis of cancer patients with PE, (iii) present the different treatment options, and (iv) highlight the actual unmet needs in PE in the landscape of cancer-associated thrombosis.

## 2. Epidemiology

### 2.1. Demographic Characteristics

Many clinical studies and reviews have assessed the risk factors of cancer patients to develop VTE, and only a few studies have focused on the PE risk. Several studies demonstrated that cancer patients with PE are generally older than noncancer patients with PE [3,4]. If the history of VTE is an important risk factor for recurrence apart from cancer (ESC/ERS), this is less certain than in cancer patients, it is as important as in patients with no cancer. Interestingly, cancer patients with a history of VTE before cancer diagnosis did not have an increased risk for cancer-associated PE [5,6] in several studies. The same idea is suggested by the absence of this item in the prediction models of risk of the first CAT or recurrent CATs. In 2009, Khorana et al. reported that patients are at high risk of VTE in the period immediately after the cancer diagnosis [7]. This is most likely caused by the introduction of chemotherapy or by the cancer-specific thrombogenicity, such as the development of metastasis [8].

**Figure 1:**
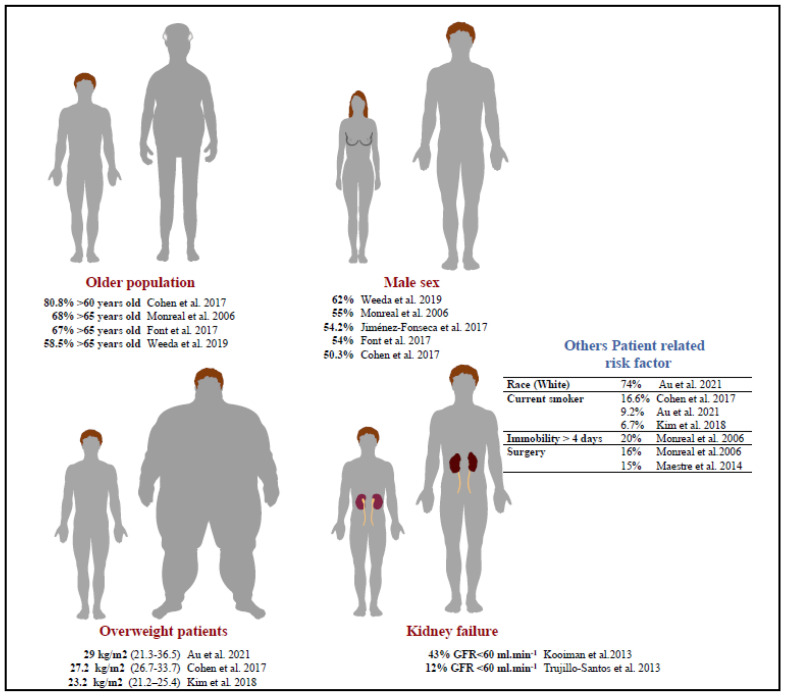
Patient-related risk factors for cancer-associated PE [4,5,6,9,10,11,12,13,14,15].

Cancer-related PE is more frequent in men [4,9,10,11,12]. The patients’ characteristics that increase the risk of PE in cancer patients are summarized in Figure 1.

**Figure 2:**
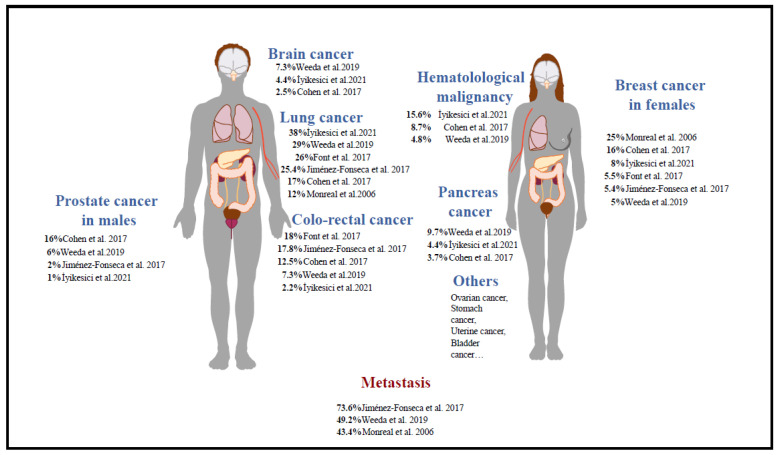
PE incidence among cancer sites associated with PE [4,9,10,11,12,16].

The repartition of the cancer site in cancer-associated PE from different studies is represented in Figure 2. İyikesici et al. investigated the clinical characteristics and CT scan findings of patients who underwent CT scans for suspected PE [16]. The EPIPHANY registry used by Weeda et al., Font, and Jiménez-Fonseca et al. was designed to assess the clinical characteristics and outcomes of patients with cancer-associated (unsuspected) PE [10]. The RIETE registry was designed to assess all types of VTE [17]. While these three studies are based on declarative data, the Cohen et al. study is based on administrative data from medical records. It is possible that strategies for seeking occult cancer in PE patients may vary widely among different regions and among clinicians and patients. However, we did not find data supporting this presumption. One has to note that the international guidelines call for no exhaustive screening since several years [18]. The most prevalent cancer site is the lung, followed by colorectal cancer. However, these cancer sites are not the most prothrombotic as compared to, for instance, pancreatic cancer. This is most likely explained by the larger number of patients with lung or colorectal cancer than of those with a more prothrombotic cancer type. The risk of CAT is largely influenced by the underlying type of cancer. Several studies have been performed to evaluate the VTE risk of different cancer types, such as Khorana et al. and the CAT cohort study [19,20]. Moik et al. summarized several studies and found that the highest thrombotic risk is observed in pancreas and stomach cancers and in primary brain tumors [21].

The presence of metastasis is also considered a risk factor of PE [22]. It has been shown that certain chemotherapies or radiotherapies increase the risk of VTE in, for instance, lung cancer, but currently no data supports that these two treatments increases PE risk [4,23].

The relationship between cancer and thrombosis and the influence of chemotherapy were taken into account by Khorana et al. [19] to create the first risk assessment model for VTE in cancer patients. Interestingly, this model integrates cancer characteristics (primary cancer site), treatment characteristics (chemotherapy prescription), patient characteristics (body mass index, BMI), and biomarkers. This integrative approach is the basis for the development of all models attempting to refine the predictive value of the Khorana score.

In 2015, Carrier et al. published a multicenter randomized trial to assess the efficacy of an exhaustive screening strategy to detect occult cancers in patients with unprovoked VTE, which included abdominal and pelvis CT scans [24]. The exhaustive screening strategy did not appear to improve the detection of occult cancers. Of the 854 patients who underwent randomization, a nonsignificant difference in additional cancer diagnoses was found using this method (19 of the 423 patients versus 14 of the 431 patients in the limited screening; *p* = 0.28).

Around the same time period, French investigators from the INNOVTE network assessed the efficacy of a screening strategy making use of 18F-FDG PET/CT scans [25]. As for the study based on CT scans, making use of 18F-FDG PET/CT scans to detect occult cancers in 200 patients with unprovoked acute VTE failed to demonstrate a positive impact on patient survival. 

In 2017, a meta-analysis of van E’s et al. computed studies that investigated different types of screening strategies, such as limited screening, the CT scan-based extensive strategy, and the PET-CT-based screening-based extensive strategy, found a higher prevalence of cancer in patients who initially had extensive screening than in those who had limited screening (OR = 2.0 95% CI (1.2–3.4), however, this difference disappeared 12 months after the diagnosis of VTE (OR = 1.4 95% CI (0.89 to 2.1) [26].

Thus, to date, according to international guidelines, it is recommended that patients with unprovoked VTE should only undergo a limited cancer screening, including thorough medical history and physical examinations, basic laboratory investigations, chest X-rays, and age- and gender-specific cancer screenings [27].

### 2.2. Demographic Characteristics

The risk of fatal PE depends on the gravity of the PE and on the patient’s characteristics, such as the cancer type and fragility. 

The mortality rate of acute PE is significantly higher in cancer patients as compared to noncancer patients (19.6% vs. 3.2%, *p* < 0.001) [3]. Within the RIETE registry, a prospective registry monitoring more than 100,000 patients in multiple medical centers around the world, PE-diagnosed cancer patients were followed from the first 3 months to 1 year after the diagnosis. A total of 4.5% of all cancer-associated thrombosis patients (188/4125) had fatal PE, including 129 individuals with initial PE and 59 with recurrent PE during the first 3 months in this study [17,28]. During the follow up, only three additional patients died of recurrent PE. These results are consistent with the study of Gussoni et al. from 2013, which presented a rate of 3.0% (97/6075) of PE-related deaths during a 3-month follow-up [29].

Shalaby et al. reported on the overall (all-cause) mortality in cancer-associated PE vs. cancer patients without PE. In this study, the authors demonstrated that the diagnosis of PE in hospitalized cancer patients was associated with a 90% increase in all-cause mortality [30]. In cancer patients, the risk of death was significantly higher during the first 3 months after PE diagnosis, which may represent the clinical deterioration after PE or that the presence of thrombosis was associated with a more advanced cancer [31]. According to Alotaibi et al., 40% of PE-diagnosed cancer patients will die within one year (60% chance of survival, 95% CI (57–64%)) and 61% within 5 years (39% chance of survival, (95% CI (36–43%)) [32]. 

In a recent autopsy study including almost 10,000 cancer patients, Gimbel et al. reported a non-neglectable and stable over time proportion of cancer patients with PE using autopsies from 2008 (incidence = 11.7%, 95% CI (10.0–13.7)) to 2019 (incidence = 15.1%, 95% CI (11.9–18.7)) [33]. These data highlight that the diagnosis of PE in cancer patients remains extremely challenging and that the rate of fatal PE is severely underestimated [33,34,35,36,37]. The real rate of PE ranges from 10 to 35% and most likely depends on the type of cancer, with a higher risk in pancreatic, gynecological, lung, and biliary cancers [33,35]. Interestingly, the reported rate of cancer-related fatal PE in Europe is higher than in Japanese or American autopsy studies, possibly due to ethnic differences. Several studies based on autopsy series are summarized in Table 1. Most studies have reported on the prevalence of PE during the autopsy. Only Svendsen et al., Ogren et al., and Valerio et al. have reported the diagnosis of PE based on the clinical setting, death certificates, or administrative data [34,36,37]. Ogren et al. clearly differentiate between PE as the primary cause of death or not [36].

Other prevalences were based on the finding of PE at the autopsies. Different definitions for PE-related death are used, and this may contribute to the broad range of reported VTE case-fatality rates. In 2020, the ISTH-established SSC on the predictive and diagnostic variables in thrombotic disease proposed a classification comprised of three categories: category A: PE-related death, category B: undetermined cause of death, and category C: cause of death other than PE [38]. This definition is used by Valerio et al. [37].

### 2.3. Risk of Recurrent PE in Cancer Patients

Patients with cancer that have developed VTE are at a high risk of recurrence, which is even increased when the first event was a PE (OR = 10.5, 95% CI (9.3–11.7) [4]. For this reason, international guidelines recommend maintaining the anticoagulant treatment for secondary thromboprophylaxis for as long as the cancer is considered active [39]. Cancer is considered active when at least one of the following conditions is met: (i) the patient has received a potentially noncurative treatment of his cancer (particularly in the case of so-called palliative chemotherapy), (ii) the follow-up shows recurrence or progression of the cancer under treatment, or (iii) the cancer treatment is ongoing [40]. Unfortunately, even under secondary thromboprophylaxis, recurrent VTE in cancer patients is frequent. The TESEO registry (NCT03855592) is a Spanish registry created in 2018 to detect emerging epidemiological trends in cancer patients and to analyze real-world clinical practice outcomes. In this registry, the 12-month cumulative incidence of recurrent VTE under anticoagulation therapy was as high as 7.1% in advanced cancers [22].

Predicting the individual risk for recurrent PE in cancer patients is a major objective in the management of cancer patients. To predict the issue of recurrent VTE (PE and/or deep-vein thrombosis, DVT) in cancer patients, the Ottawa risk assessment model was developed as a specific risk assessment model. This risk assessment model is a clinical prediction rule that stratifies the risk for VTE recurrence in patients with cancer and VTE by using simple predictors such as (female) sex (+1), lung cancer (+1), breast cancer (−1), cancer stage (−2), and prior history of VTE(+1) [41]. During the first six months of anticoagulation, the sum of the points assigns a low (score ≤ −1), intermediate (score = 0), or high (score ≥ 1) risk of recurrent VTE to patients with recurrence rates of 5.1%, 9.8%, and 13.9%, respectively [41]. Based on a meta-analysis of nine studies (in total, 14,963 cancer patients with VTE), Delluc et al. considered the Ottawa score and its modified version as useful tools to stratify the 6-month risk for VTE recurrence in patients on anticoagulation [42]. In the meta-analysis, the original score classified 49.3% of the patients into the high-risk group with a good sensitivity of 71%, while the modified score was more suitable to classify patients into the low-risk group (19.8% with a sensitivity of 92%) [42]. In 2021, the PREDICARE study assessed the performance of the Ottawa score in a prospective multicentric cohort study [31]. The Ottawa score performed relatively poorly, with an area under the curve of 0.60 (95% CI (0.55–0.65)). Moreover, in the multivariable analyses, none of the potential risk factors for recurrent VTE were associated with recurrent VTE after 6 months [43]. Therefore, the PREDICARE study concluded that currently no reliable risk assessment model for recurrent VTE including PE in cancer patients is fit to be used in daily practice.

## 3. Diagnosis and Prognosis of PE in Cancer Patients

### 3.1. Symptomatic PE in Cancer Patients

**Figure 3:**
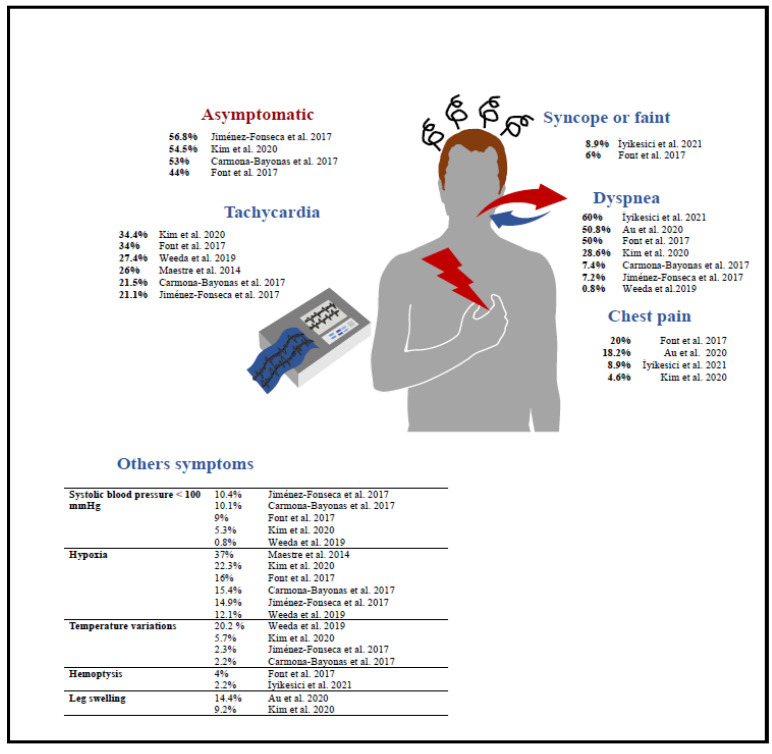
Repartition of the symptoms in cancer-related PE [5,6,10,11,12,15,16,22].

In cancer patients, PE presentation is less symptomatic and is commonly with nonspecific symptoms possibly related to the cancer itself [44]. The frequency of the symptoms as reported in the literature are summarized in Figure 3 [45].

To improve the diagnosis of low-probability PE, clinical prediction rules have been developed. Clinicians rely on prediction rules such as the Wells or Geneva rules. These rules incorporate plasma D-dimer measurements, which are recommended in outpatients/emergency department patients with a low or intermediate clinical probability or those that are PE-unlikely. In these same prediction rules, cancer is already a predictor that increases the likelihood that patients will undergo a redundant computed tomography pulmonary angiogram (CTPA) for PE and will not be tested for D-dimer levels [46,47]. Making use of different methods to test D-dimer levels demonstrated that cancer patients have a higher D-dimer concentration, regardless of the presence of VTE [48]. This results in a lower specificity of D-dimer tests in cancer patients [49]. Following the recommendations of the ADJUST-PE study, the age-adjusted increased cutoff for D-dimer interpretation in the general VTE population became the standard [50]. In 2017, Wilts et al. performed an analysis of the cancer patients included in the ADJUST-PE study [51]. Here it was shown that the age-adjusted D-dimer cutoff decreased the need of performing CTPA to rule out PE in cancer patients (9.9% CTPA with the age-adjusted cutoff vs. 19.7% CTPA with the conventional cutoff 0.5 μg/mL, out of 429 included cancer patients, *p* < 0.001). Nowadays, the age-adjusted D-dimer is the standard also in cancer patients. YEARS is a newly developed clinical prediction rule in which the D-dimer cutoff was dependent on the present YEARS items (DVT, hemoptysis, and PE as the most likely diagnosis) [52]. Nevertheless, this approach was not completely safe as compared to the traditional or the age-adjusted cutoff, and it showed a failure rate of 2.6% (95% CI 1.3–5.2) in the subgroup of 336 included cancer patients. A cancer patient-dedicated diagnostic management randomized trial, the Hydra study, to compare the safety and efficiency of the YEARS algorithm to the safety and efficiency of CTPA alone is currently ongoing [53].

### 3.2. Unsuspected PE in Cancer Patients

Unsuspected cancer-associated PE can be detected in patients that undergo CTPA but also PET-CT [54]. The EPIPHANY registry is a prospective observational registry that describes the clinical characteristics and outcomes of patients with all cancer-associated PE, including unsuspected events of patients with symptoms not suggestive of PE and patients with no symptoms (truly asymptomatic). Based on this registry, unsuspected PE represents 58% of all PE events, and truly asymptomatic unsuspected PE represents 31% [10]. The first data based on retrospective studies in cancer patients suggested a morbi-mortality of unsuspected PE and truly asymptomatic unsuspected PE comparable to that of symptomatic PE [55,56]. In the prospective study of Kraaijpoel et al. dedicated to unsuspected PE in cancer patients, recurrent VTE after unsuspected PE occurred in 6.0% of the patients (95% CI (4.4–8.1%)) [57]. In 2019, Chang et al. showed that anticoagulant treatment significantly improved the overall survival of patients with proximal unsuspected PE (main or lobar level of pulmonary arteries, median survival 12.2 vs. 23.4 months, *p* = 0.023), but not in patients with distal unsuspected PE (segmental or subsegmental level of pulmonary arteries, median survival 21.2 vs. 15.1, *p* = 0.906) [58]. If unsuspected PE is common in cancer patients, luckily, it are not as prognostically important as symptomatic PE [56,57,59,60,61,62,63,64,65,66,67,68,69,70,71,72,73,74]. Several studies on the incidence of death rate, recurrent VTE, bleeding, and sequel in unsuspected PE are summarized in Table 2. As shown in Table 2, the overall survival varies a lot between different studies. Unfortunately, adequate evidence such as a well-designed meta-analysis is lacking, and no conclusions can be drawn on whether the survival has improved in the recent years or not.

### 3.3. Subsegmental PE in Cancer Patients

The incidence of subsegmental PE is reported as high, at 30% of all forms of PE [75]. In 2010, Carrier et al. realized a large meta-analysis of 22 studies and suggested that in noncancer patients, the diagnosis of isolated subsegmental PE has no major impact on the patient outcome [76]. Based on these results, in standard practice, isolated subsegmental PE is not always treated. However, patient outcome after subsegmental PE diagnosis seemed different in a cancer population. Leroux et al. performed a retrospective analysis of cancer patients referred for CTPA or ventilation/perfusion lung scintigraphy for suspicion of acute PE [77]. During a period of 10 years, 2345 cancer patients were included with a PE rate of 16% (373/2345) [75]. Among them, 122 patients had a solitary subsegmental or one single-segmental PE. Although, within the first year, these 122 patients presented a similar survival rate as those without PE; after 1 year, the overall survival declined to approximate that of cancer patients with proximal PE [75]. The current consensus guidelines recommend the anticoagulation treatment of cancer patients with subsegmental PE, even if incidentally detected [78,79,80].

### 3.4. Central Venous Catheter-Related Pulmonary Embolism in Cancer Patients

In the MEGA study, in a Dutch population-based case control study, one out of three upper-limb DVTs is complicated by PE [81]. In addition, there is a high incidence of upper-limb DVT in cancer patients that is favorized by the presence of a central venous catheter [81]. In the ONCOCIP study, a prospective multicenter cohort study of 3032 patients with a solid tumor and a central venous catheter, around 1 out of 20 central venous catheter-related thrombosis cases were complicated by PE [82]. In a study from Blom et al., cancer patients with a central venous catheter showed an 18-fold increased risk for PE, as compared to noncancer patients with a central venous catheter [8].

When PE is secondary to central venous catheter thrombosis, the duration of the anticoagulant treatment is 3 months, instead of the usual 6 months, in the case of an isolated or a lower-limb DVT associated with PE. The RIETE registry demonstrated that for catheter-related thrombosis, the rate of recurrent VTE during the anticoagulant treatment (2.83 per 100 patient years) and after the 3 months of treatment (2.88 per 100 patient years) is relatively low as compared to non-catheter-related thrombosis in cancer patients [83]. In this study, with multivariable analyses, several risk factors for recurrent VTE, such as the initial presentation with PE or kidney failure, were identified during the follow-up [83]. The initial presentation with PE significantly increased the risk of recurrent DVT (OR, 4.90; 90% CI (1.58–15.2)), while for recurrent PE, no statistical difference was found [83].

### 3.5. How to Assess the Risk of Mortality in PE Cancer Patients

Within the general VTE population, prognosis stratification of PE is commonly assessed with the (simplified) pulmonary embolism severity index ((s)PESI) and the hemodynamic tolerance of PE. The (s)PESI cancer patients are systematically classified in the “not low risk” categories, and this suggests that we should reconsider the utility of the (s)PESI for cancer patients. The COMMAND VTE registry was a multicenter registry enrolling consecutive patients with acute symptomatic VTE. In this registry, 368 cancer patients with PE were enrolled [84]. The cumulative incidences of mortality and PE-related death for the 30 first days were lower in patients with a (s)PESI score of 1 than in patients with a (s)PESI score ≥ 2 (6.3% vs. 13.1%, log rank *p* = 0.03, and 0.7% vs. 3.9%, log rank *p* = 0.046) [84]. Unfortunately, the (s)PESI failed to reliably predict short-term recurrent VTE and major bleedings [84]. Within the POMP-C study, a risk assessment model was developed to predict mortality among ambulatory cancer patients with PE within 30 days after the PE diagnosis. The performance of this model was compared to PESI [55]. In the POMP-C study, the PESI score confirmed a low performance in cancer patients (area under the curve (AUC) of 0.68, 95% CI (0.6–0.76)) as compared to noncancer patients (AUC of 0.79, 95% CI (0.75–0.83) [55]. Parameters that were kept among the risk predictors found in the cancer population-based analysis to integrate the POMP-C risk assessment model were an altered mental status, body weight, a heart rate over 99 beats/min, respiratory distress, an increased respiratory rate, the presence of unilateral limb swelling, and a “do not resuscitate” status [55]. The POMP-C risk assessment model demonstrated promising results in the derivation dataset (AUC cancer patients = 0.84, 95% CI (0.78–0.89)) and in the validation sample (AUC cancer patients = 0.86, 95% CI (0.78–0.93)). Unfortunately, until now, it has never been validated in a large prospective study. Not being able to assess the early prognosis of cancer-related PE has consequences. Cancer patients are still not eligible for ambulatory management, and cancer diagnosis is still an exclusion criterion in clinical trial testing for more aggressive therapeutic options such as systemic thrombolysis.

## 4. Management of Cancer Patients with PE

### 4.1. Anticoagulation

Within the cancer patient population, regarding the indication of anticoagulation, no differences are made on whether the patient has developed a PE or a non-catheter-related DVT. Cancer patients are always treated for at least 6 months, and if the cancer is still considered as active after this period (patient under treatment, patient with detectable cancer mass), the treatment is extended. Managing VTE in cancer patients is more challenging as compared to noncancer patients because of the frail balance between the increased risk of recurrent VTE and the increased risk of major bleeding due to the anticoagulant treatment. In 2003, the CLOT trial guidelines endorsed low molecular weight heparin (LMWH) monotherapy as the standard-of-care treatment for VTE cancer patients [85]. With the arrival of the new generation of anticoagulants, the direct oral anticoagulants (DOACs), several randomized controlled trials were launched to assess the security and safety of this new therapeutic class in the initial treatment of VTE in cancer patients, and all studies concluded that DOACs are favorable for thrombosis treatment over LMWHs [86,87,88,89,90,91].

In the Planquette et al. meta-analysis, five randomized controlled trials comparing DOACs and LMWHs (Hokusai VTE cancer, ADAM-VTE, SELECT-D, CASTA DIVA, and CARAVAGGIO) were included [91]. This meta-analysis concluded that DOACs were more efficient to prevent VTE, while also, an increased risk of clinically relevant bleeding with DOACs was pointed out [91]. Further research is needed to assess the external validity of these randomized controlled trials on everyday practice in which unselected patients display more comorbidity. Nowadays, more and more cancer patients benefit from DOACs as the first option for anticoagulation for a (expected) better compliance and a more practical use (pills over injection), as long as their bleeding risk allows for it. 

Several limitations on the use of DOACs exist. According to international guidelines for patients with PE and cancer, DOACs should be considered as an alternative to LMWHs in patients without a high risk of bleeding [38,78,80]. Thus, in gastrointestinal cancer and urinary tract cancer, DOACs should be avoided. Patients with primary brain cancer or brain metastasis, patients with severe thrombocytopenia or anemia, and patients with a short life expectancy were almost always excluded from the clinical trial on DOACs. Moreover, there is also no reliable knowledge on the drug–drug interaction between DOACs and some cancer treatments [38,78,80].

### 4.2. Other Available Treatments of Pulmonary Embolism in Cancer Patients

In the general VTE/PE patient population, the guidelines approve other treatments for PE such as inferior vena cava filters (IVCF), as well as systemic thrombolysis, percutaneous catheter direct treatment, and surgical embolectomy for PE embolisms. The guidelines recommend the use with caution of IVCFs in cancer patients with a high risk of bleeding. The other interventional procedures are not recommended in cancer patients. 

Systemic thrombolysis is a very effective VTE treatment in noncancer patients at the cost of an increased hemorrhagic risk, in particular, intracranial bleeding. Therefore, intracranial cancers are considered as a contradiction for thrombolysis. Few studies exist on thrombolysis in cancer patients, and administrative data enabled the gathering of more information on a bigger sample size. Weeda et al. in 2019 and Shalaby et al. in 2021 used the same national inpatient sample database to assess the use of thrombolysis in cancer patients, and both studies found that less cancer patients received thrombolysis as compared to the noncancer population (OR = 0.55, 95% CI (0.48–0.64), and OR = 0.68, 95% CI (0.64–0.72), respectively), particularly in individuals with metastatic disease [12,30]. Unfortunately, no data on the incidence of bleeding or mortality was reported in these two studies. Jara-Palomares et al. and Iskandar et al. reported divergent risks for major bleeding during the thrombolysis procedure: a significatively increased risk for Jara-Palomares et al. (OR = 2.1, 95% CI (1.1–3.9)) and a non-significant risk for Iskandar et al. (OR = 0.98, 95% CI (0.55–1.78)) [92,93]. The underuse of systemic thrombolysis due to the fear of provoking bleedings has led to a less accurate estimation of the risk of bleeding in cancer patients who undergo thrombolysis. 

The IVCF is only used as an antithrombotic strategy in patients that cannot be treated with anticoagulants because of its inferior efficacy and the risk of filter-associated DVT [40]. This guideline was established based on the PREPIC2 trial, a trial that did not include a subset analysis of cancer patients [94]. Small prospective and retrospective studies were performed on consecutive IVCF cancer patients, patients with a particularly high risk of bleeding (brain tumor) or with a high risk of VTE recurrence (advanced stage of cancer), all with divergent results [95,96,97,98,99,100]. Regarding studies based on administrative databanks, the data converge towards a low risk of death but diverge on the risk of recurrent DVT. To summarize, IVCF placement appears safe in cancer patients, although the putative increased risk of DVT should be explored prospectively. The incidence of recurrent VTE and mortality after IVCF insertion is summarized in Table 3.

To our knowledge, only Edupuganti et al. studied the incidence of the retrieval of IVCF cancer patients [105]. Here it was demonstrated that only 7.5% of cancer patients obtained an IVCF retrieval [105].

Current guidelines reserve catheter-directed therapy for patients who fail to respond to anticoagulant therapy. Three key studies investigated PE treatment using catheter-directed therapy, a randomized controlled trial (the ULTIMA study) and two prospective cohort studies (the SEATTLE II and the PERFECT studies) [106,107,108]. A small number of patients with cancer were included, and the outcome of that specific subgroup was not assessed. For this reason, clinically relevant data on the efficacy, safety, and indications of catheter-directed therapy are lacking in cancer patients.

## 5. What Are the Remaining Unmet Needs in Cancer Patients with PE?

Throughout this review, we have addressed various issues in the management of cancer-associated PE that are currently unsolved, such as the assessment of the low PE diagnosis probability, the prediction of adverse events, and the PE prognosis assessment. Other issues that still need to be settled are how to predict the risk of bleeding, what is the best secondary prevention to adopt, and how to detect post-PE syndrome.

### 5.1. How to Predict Better the Bleeding Risk in Cancer Patient with PE?

The two most commonly prescribed anticoagulant drugs in cancer patients are DOACs and LMWHs. [109] In the CARAVAGGIO study, major bleeding was more frequent in cancer patients with PE than in cancer patient with DVT, treated either with apixaban (68.2% vs. 44.8%) or LMWHs (56.5% vs. 49.6%) [110]. These results emphasize that correct assessment of the bleeding risk of cancer patients is crucial. In our recently published systematic review, we have shown that out of the 15 available risk assessment models, none were validated for the bleeding risk in cancer patients [111]. The CAT-BLEED risk assessment model, recently developed specifically for cancer patients with VTE, seems promising, but has not been externally validated yet [112]. The inclusion of biomarkers in the risk assessment models may increase their performance. New drugs with a lower anticoagulant potency, such as FXI inhibitors, are promising to reduce bleeding events in cancer patients [113].

### 5.2. What Is the Best Secondary Prevention for the Management of PE in Cancer?

Using anticoagulant-associated bleeding risk assessment models, a tailored anticoagulant treatment for patients based on their risk of bleeding, seems to be the future of treating cancer-associated thrombosis. Under certain conditions of unprovoked VTE in the general population, a reduced-dose regimen of DOACs is possible to prevent secondary VTE, and it is of interest to explore this option for cancer-associated thrombosis. The aim of the API-CAT trial is to assess whether, in cancer patients, a reduced-dose regimen of the FXa inhibitor, apixaban, is non-inferior to a full-dose regimen of apixaban, since in noncancer patients, the reduced-dose regimen is already considered safe and efficient [114]. The results of this study are expected in 2024.

Another approach to decrease the risk of bleeding could be to make use of a new and safer anticoagulant, able to prevent recurrent PE, with a lower risk of bleeding than with the use of DOACs. FXIa inhibitors are newly developed drugs that have proven their effectiveness in the prevention of VTE after orthopedic surgery [115]. Abelacimab, an antibody against FXIa, is going to be tested soon in two phase III studies (NCT05171049, NCT05171075) in patients with cancer-associated thrombosis.

### 5.3. How to Screen for Post-PE Syndrome in Cancer Patients?

Because of the advances in medical treatment and drugs, cancer patients have an increased chance of survival. Therefore, the optimal follow-up of cancer patients deserves the same concern as any other VTE patient. Recently, the European Society of Cardiology in collaboration with the European Respiratory Society realized a list of recommendations to follow up PE. Monitoring lung function to detect (objective of subjective) chronic impairments as a clue of post-PE syndrome was recommended as one of the key aspects of PE management [116]. The most severe form of post-PE syndrome is chronic thromboembolic pulmonary hypertension (CTEPH). Catella-Chatron et al. reported on post-PE syndrome and CTEPH assessment in cancer patients [117]. They observed that the medical evaluation may be lower for patients with cancer, despite that some of them have a reasonable survival expectancy [117]. Within their meta-analysis they also showed that the incidence of CTEPH is the same in cancer and noncancer patients, although screening for CTEPH in cancer patients is less common [117]. This might be explained by the non-specificity of the CTEPH clinical signs that often coincide with the clinical signs of the cancer [117].

## 6. Conclusions

Substantial progress has been made on the treatment of PE in cancer patients in the last couple of decades, however, further studies are required to evaluate and improve the management of this frail patient population. Because PE in cancer patients is pleomorphic, its prevention, diagnosis, and prognosis remain complicated to assess. Cancer, especially metastatic cancer, is associated with an increased bleeding risk. This bleeding risk is difficult to assess, and the use of aggressive interventional therapies or DOACs in high-risk patients is currently discouraged. Moreover, with the improvement of life expectancy in cancer patients, a rise of awareness to follow up thrombotic events is necessary.

## Figures and Tables

**Table 1:** Fatal cancer-related PE in autopsy series.

Authors	Number of Cancer Patients	Number of PE-Related Deaths, %	
Svendsen et al. [34] 1989 (Europe)	6197	648	10.5%
Sakuma et al. [35] 2006 (Japan)	65,181	1514	2.3%
Ögren et al. [36] 2006 (Europe)	23,796	2369 5448 *	10.0% 28.9% *
Gimbel et al. [33] 2021 (Europe)	9571	1191	12.4%
Valerio et al. [37] 2021 (USA)	127,945	Category A ISTH 209 Category B ISTH 6571	0.2% 5.1%

* PE present at the autopsy, category A ISTH: PE-related death, category B ISTH: undetermined cause of death.

**Table 2:** Incidence of death rate, recurrent VTE, bleeding, and sequel in unsuspected PE.

Authors	N	Follow-Up (Month)	Death	Recurrent VTE	Bleeding	Sequel	Therapeutic Anticoagulation
Sun et al. [59] 2010	113 *	9.3	50%	/	/	/	45%
Den Exter et al. [60] 2011	51	3	27.5%	/	3.9%	/	100%
		6	35.3%	/	5.9%	/	
		9	43.1%	/	5.9%	/	
		12	43.1%	/	5.9%	/	
Abdel-Razeq et al. [61] 2011	34	1	26.5%	5.9%	/	5.9%	85%
O’Connell et al. [62] 2011	96	2	16.7%	/	/	/	84%
		6	33.3%	/	/	/	
		12	62.5%	/	/	/	
O’Connell et al. [63] 2011	21	12	47.6%	/	/	/	/
		24	57.1%	/	/	/	/
		36	66.7%	/	/	/	/
		48	76.8%	/	/	/	/
		60	100	/	/	/	/
Dentali et al. [64] 2011	60	6	45%	/	/	/	93%
Sahut D’Izarn et al. [65] 2012	66	6	17%	6%	4%	/	100%
van Der Hulle et al. [66] 2016	926	6	37%	5.8%	4.7%	/	79%
Peris et al. [67] 2016	715	12	20.1%	28%	6.9%	/	98%
Myat Moe et al. [68] 2018	26	2	11.5%	/	/	/	88.5%
		7	50%	/	/	/	
Bozas et al. [69] 2018	234	1	3.4%	/	/	/	/
		3	15%	/	/	/	
		6	31%	2.6%	2.1%	/	
Ahn et al. [70] 2018	258	1	7.8%	<1%	1.9 %	/	96.1%
Chang et al. [58] 2019	474	5.6	50%	/	/	/	52.3%
Kraaijpoel et al. [57] 2019	695	12	41%	5.9%	5.6%	/	96.2%
Muñoz Martín et al. [71] 2020	25	1 3	0%	/ 4%	/ 4%		100%
Qdaisat et al. [72] 2021	904	0.25	1.8% 9.9% 22.1%	/	/	/	92.5%
		1		/	/	/	
		3		/	/	/	
Maraveyas et al. [73] 2021	695	12	41%	5.9%	5.7%		97%
Peris et al. [74] 2021	946	3	11%	1.6%	3.2%	/	>95%

* Lung cancer patients only.

**Table 3:** Incidence of death rate and recurrent VTE post-IVCF insertion.

	Study Design	Population of Non-Prophylactic IVCF	N	Recurrent VTE	All-Cause Mortality
Olin 1987 [95]	Comparative nonrandomized Monocentric study	Brain cancer patients at high risk of bleeding	24	4% in the IVCF group	28% in each group
Cohen 1991 [96]	Comparative nonrandomized Monocentric study	Cancer patients	18	0% in the IVCF group	/
Cohen 1992 [97]	Cohort study	Cancer patients	41	2.4%	56%
Hubbard 1994 [98]	Cohort study	advanced malignancies patients	31	19.4%	/
Schwarz 1996 [99]	Cohort study	Cancer patients	182	6.6%	0%
Greenfield 1997 [100]	Registry study	Cancer patient followed for their risk of recurrent cancer	166	36% recurrent VTE of the 86 patients presenting a recurrent cancer	69.7% of the 166 IVCF
Barginear 2012 [101]	Randomized control study	Cancer patients	32	3.1%	0%
Mismetti 2015 [94]	Randomized control study	Hospitalized patients with PE and DVT	33	3%	/
Brunson 2016 [102]	Cohort study	Hospitalized cancer patients	2747	Risk of VTE HR = 0.81, 95% CI (0.6–1.08)	Risk of short-term mortality HR = 1.12, 95% CI (0.99–1.26)).
Stein 2018 [103]	A population-based cohort study using administrative data	Hospitalized patients with PE	6589	/	8.1%
Balabhadra 2020 [104]	A population-based cohort study using administrative data	cancer patients with a diagnosed VTE	33,740	HR = 0.69; 95% CI, 0.64–0.75; *p* < 0.001	/

## Data Availability

Not applicable.
